# Prevalence of dyslipidaemia among HIV-infected patients receiving combination antiretroviral therapy in North Shewa, Ethiopia

**DOI:** 10.1371/journal.pone.0250328

**Published:** 2021-04-27

**Authors:** Temesgen Fiseha, Worku Alemu, Henok Dereje, Zemenu Tamir, Angesom Gebreweld

**Affiliations:** 1 Department of Clinical Laboratory Science, College of Medicine and Health Sciences, Wollo University, Dessie, Ethiopia; 2 Department of Medical Laboratory Sciences, College of Health Sciences, Addis Ababa University, Addis Ababa, Ethiopia; 3 Department of Medical Laboratory Science, College of Health Sciences, Mekelle University, Mekelle, Ethiopia; Università degli Studi di Milano, ITALY

## Abstract

**Background:**

Data on the burden of dyslipidaemia among people with HIV undergoing antiretroviral therapy (ART) in sub-Saharan Africa are limited and little is known about the factors contributing for poor lipid profiles. The aim of this study was to determine the prevalence of dyslipidaemia and factors associated with lipid levels among HIV-infected patients receiving first-line combination ART in North Shewa, Ethiopia.

**Methods:**

A cross-sectional study was conducted between April and December 2018 among 392 HIV-infected adults receiving first-line ART for at least six months at the ART clinic of Mehal Meda Hospital in North Shewa, Ethiopia. Blood samples were collected for determination of total cholesterol (TC), triglyceride (TG), high-density lipoprotein cholesterol (HDL-c), low-density lipoprotein cholesterol (LDL-c), and CD4 counts. Logistic regression analysis was used to determine factors associated with lipid abnormalities.

**Results:**

The prevalence of dyslipidaemia was 59.9% (95% CI 55.0–64.7%). High TC, high TG, low HDL-c, and high LDL-c were obtained in 47.3%, 30.9%, 19.4% and 29.6%, respectively. Fifty-four participants (13.8%) had high ratio of TC/HDL-c (TC/HDL-c ratio ≥ 5). Older age was independently associated with high TC (AOR = 2.51, 95% CI 1.64–3.84), high TG (AOR = 2.95, 95% CI 1.85–4.71), low HDL–c (AOR = 2.02, 95% CI 1.17–3.50), and high LDL–c (AOR = 3.37, 95% CI 2.08–5.47). Living in an urban area (AOR = 2.61, 95% CI 1.16–6.14) and smoking (AOR = 3.61, 95% CI 1.06–12.34) were associated with low HDL–c. Participants with longer duration of ART use were more likely to have high TG (AOR = 1.86, 95% CI: 1.13–3.07), low HDL–c (AOR = 3.47, 95% CI: 1.75–6.80), and high LDL–c (AOR = 2.20, 95% CI 1.30–3.71). High BMI was independently associated with higher TC (AOR = 2.43, 95% CI 1.19–4.97), high TG (AOR = 4.17, 95% CI 2.01–8.67) and high LDL–c (AOR = 6.53, 95% CI 3.05–13.98).

**Conclusions:**

We found a high prevalence of dyslipidaemia among HIV-infected patients receiving first-line ART in North Shewa, Ethiopia. There is a need for monitoring of blood lipid levels in patients with HIV on long term first-line ART with a special attention to be focused on older age, urban residents, longer duration of ART use, high BMI and smokers.

## Introduction

The introduction of combined active antiretroviral therapy (ART) for human immunodeficiency virus (HIV) infection has dramatically reduced morbidity and mortality from AIDS-related causes and increased the life expectancy of HIV-infected people [1]. With improved life expectancy and prolonged survival of people living with HIV, metabolic (dyslipidaemia, insulin resistance, and diabetes) and cardiovascular complications are being observed with increasing frequency among these patients with access to ART [2, 3]. Dyslipidaemia is a well-recognized complication of combination ART; occurring in up to 70%–80% of HIV-infected subjects who are receiving combined ART and is mainly associated with specific antiretroviral agents of the nucleoside reverse transcriptase inhibitors (NRTIs), nonnucleoside reverse transcriptase inhibitors (NNRTIs) and protease inhibitor (PI) classes [3–6].

Dyslipidaemia associated with combination ART use is characterized by increased levels of serum total cholesterol (TC), low-density lipoprotein cholesterol (LDL-c) and triglycerides (TG) and a decreased high density lipoprotein-cholesterol (HDL-c) level [3, 5, 6]. These changes in lipid level occur early after the initiation of combined ART, and have been associated with an increased risk for cardiovascular disease and mortality [7–9]. In patients receiving combination ART, changes in lipid profiles have been also shown to be an independent risk factor for adverse outcomes, including cardiovascular-related events, reduced life expectancy and increased use of medical resources, which can greatly increase healthcare costs of the disease and reduce quality of life of patients with HIV [8, 10]. Assessment and early detection of these lipid changes is, therefore, crucial during ART use to facilitate the employment of intervention strategies (changes in diet and lifestyle, treatment switching, and pharmacotherapy) and to prevent adverse outcomes related to dyslipidaemia and preserve life expectancy among patients with HIV [6, 8].

There is evidence demonstrating an increased risk and prevalence of dyslipidaemia among HIV infected persons in Africa [11]. Furthermore, lipid abnormalities associated with ART use are suggested to contribute to increased cardiovascular risk among patients with HIV/AIDS in sub-Saharan Africa (SSA) [12]. Despite this, limited data are available on the burden of dyslipidaemia among people with HIV undergoing long-term ART in SSA and little is known about the factors contributing for poor lipid profiles. Such data are of great relevance to inform prevention, early detection and prompt management of cardiovascular disease risk within HIV care and treatment programs. Therefore, in this study we determined the prevalence of dyslipidaemia and factors associated with lipid levels among HIV-infected patients receiving first-line combination ART in North Shewa, Ethiopia.

## Methods

### Study setting and study population

This was a cross-sectional study conducted between April and December 2018 at the ART clinic of Mehal Meda Hospital (MMH). MMH; located in Mehal Meda town of Menz Gera Midir in North Shewa, Eastern Amhara state, Ethiopia, provides HIV/AIDS interventions including free diagnosis, treatment and monitoring. HIV-infected patients attend the ART clinic once in a month for clinical evaluation and prescription refill for combination antiretroviral (ARV) regimens. All HIV-infected patients aged 18 years or older who had been receiving first-line combination ARV regimens for a minimum of six months were consecutively enrolled. Critically ill patients, patients already on anti-dyslipidemic drugs, pregnant women, known diabetes mellitus and renal failure patients were excluded from the study. A total of 422 eligible patients visiting the ART clinic were recruited for this study. This was based on sample size calculation using 80% power, 5% level of significance, 50% prevalence and non-response rate of 10%. A total of 30 patients were excluded from this study (due to faulty laboratory results and not being fasting at the time of appointment), leading to a total of 392 patients.

### Data collection

Data on socio-demographics, medical history (including diabetes mellitus, renal failures, anti-dyslipidemic drug use), and lifestyle behaviors (smoking and alcohol consumption) were collected using a structured questionnaire which was developed in English with modification from the WHO STEPS. Clinical data including duration since HIV diagnosis, duration of ART use and types of ART-regimens were collected from patient records. Anthropometric measurements (weight and height) were taken by a trained nurse. Body mass index was calculated as weight in kilograms divided by height in meter square (kg/m^2^). Blood sample was collected from each participant in the mornings after an overnight fasting and centrifuged at 3000 cycles/ minute, and then serum was obtained for lipid profiles. Serum total cholesterol, triglycerides and HDL-c concentrations were assessed by specific colorimetric assays, using an automated analyzer system (BS-200, Shenzhen Mindray Bio-medical Electronics Co., Nanshan, China). LDL-c concentration was determined using the Friedewald et al. formula [13]. Abnormal lipid profile was defined as TC ≥ 200 mg/dl, TG ≥ 150 mg/dl, HDL-c < 40 mg/dl, LDL-c ≥ 130 mg/dl and TC/HDL-c ratio ≥ 5 in accordance with the United States National Cholesterol Education Program, Adult Treatment Panel III (NCEP-ATP III) guidelines [14]. CD4 cell count was measured using the BD FACSCOUNT system (Becton Dickenson and Company, California, USA).

### Statistical analysis

Data were entered in to “EpiData version 3.1” and exported to Statistical Package for Social Sciences (SPSS) Version 20 for analysis. Chi squared (x2) test was used for comparison of categorical variables while the Student t-test (or in case of asymmetry the Kruskal-Wallis test) was used to compare continuous variables. A multivariable logistic regression was used to determine the factors independently associated with abnormal level of each lipid profile. Adjusted odd ratios (AOR) and their 95% confidence intervals (95%CI) were also obtained. *P* value less than 0.05 was used to indicate statistical significance.

### Ethical consideration

The study was approved by the Institutional Review Board of College of Medicine and Health Sciences, Wollo University. A written informed consent was obtained from each study participants.

## Results

### Characteristics of study participants

The study included 392 patients who were attending the ART clinic of MMH for their routine prescription refill and monitoring. Of these, 256 (65.3%) were females and 136 (34.7%) were males. The mean (± standard deviation [SD]) age of patients was 41.2 ± 14.4 years (ranging from 18–80 years). The mean BMI of patients was 20.4 ± 3.1 kg/m^2^ and 40 (10.2%) were overweight/obese. Thirty-eight (9.7%) patients reported using alcohol and only 13 (3.3%) were current smokers. The mean CD4 lymphocyte count was 400.9 ± 290.9 cells/mm^3^. All patients received a triple-drug regimen including 2 NRTIs and an NNRTI, with lamivudine (3TC) constantly present in all the first-line triple combination ARV regimens. One hundred and sixty-five (42.1%) patients were using tenofovir (TDF)-based NRTIs regimens, and 215 (54.8%) were on nevirapine (NVP)-based NNRTI regimen. There were no significant differences in patient characteristics between males and females except for smoking which was significantly higher in males than in females (*P* = 0.001) (Table 1).

**Table 1:** Characteristics of study participants in North Shewa, Ethiopia.

	All participants N = 392, n (%)	Males N = 136, n (%)	Females N = 256, n (%)	P-value
Age (years)	41.2 ± 14.4	42.6 ± 14.4	40.5 ± 14.3	0.166
Residence				
Urban	316 (80.6)	109 (80.1)	207 (80.9)	0.894
Rural	76 (19.4)	27 (19.9)	49 (19.1)	
Education				
< high school	196 (50.0)	70 (51.5)	126 (49.2)	0.750
≥ high school	196 (50.0)	66 (48.5)	130 (50.8)	
Duration of HIV infection				
≤ 5years	85 (21.7)	25 (18.4)	60 (23.4)	0.303
> 5 years	307 (78.3)	111 (81.6)	196 (76.6)	
Duration on ART				
≤ 5years	147 (37.5)	47 (34.6)	100 (39.1)	0.443
> 5 years	245 (62.5)	89 (65.4)	156 (60.9)	
ARV regimen combinations				
TDF/ 3TC/NVP	89 (22.7)	30 (22.1)	59 (23.0)	0.212
TDF/ 3TC/EFV	76 (19.4)	33 (24.3)	43 (16.8)	
AZT/ 3TC/NVP	88 (22.4)	23 (16.9)	65 (25.4)	
AZT/ 3TC/EFV	68 (17.3)	21 (15.4)	47 (18.4)	
D4T/ 3TC/NVP	38 (9.7)	16 (11.8)	22 (8.6)	
D4T/ 3TC/EFV	33 (8.4)	13 (9.6)	20 (7.8)	
CD4 count (cells/mm^3^)	400.9 ± 290.9	401.8 ± 297.6	400.5 ± 287.9	0.967
< 200	120 (30.6)	45 (33.1)	75 (29.3)	0.490
≥ 200	272 (69.4)	91 (66.9)	181 (70.7)	
Smoking	13 (3.3)	11 (8.1)	2 (0.8)	0.001
Alcohol use	38 (9.7)	17 (12.5)	19 (7.4)	0.197
Body mass index (Kg/m2)	20.5 ± 3.1	20.3 ± 3.4	20.5 ± 3.0	0.464
< 25	352 (89.8)	121 (89.0)	231 (90.2)	0.727
≥ 25	40 (10.2)	15 (11.0)	25 (9.8)	

ARV, antiretroviral; TDF, tenofovir; AZT, zidovudine; D4T, stavudine; 3TC, lamivudine; EFV, efavirenz; NVP, nevirapine

### Mean lipid levels

The mean lipid levels of HIV infected adults on ART are summarized in Table 2. Mean levels of TC, TG, HDL-c, and LDL-c were 213.89 ± 61.66 mg/dL, 132.83 ± 55.91 mg/dL, 70.99 ± 25.89 mg/dL, and 116.33 ± 54.72 mg/dL, respectively, with no significant differences between males and females (*P* > 0.05).

**Table 2:** Mean lipid levels among HIV infected adults receiving ART stratified by gender.

	Total	Male	Female	P-value
TC(mg/dl), Mean ± SD	213.89 ± 61.66	216.77 ± 65.09	212.36 ± 59.84	0.501
TG(mg/dl), Mean ± SD	132.83 ± 55.91	137.03 ± 58.60	130.60 ± 54.41	0.279
HDL-c(mg/dl), Mean ± SD	70.99 ± 25.89	68.60 ± 27.09	72.27 ± 25.19	0.181
LDL-c(mg/dl), Mean ± SD	116.33 ± 54.72	120.77 ± 59.51	113.97 ± 51.96	0.242

TC, total cholesterol; TG, triglyceride; HDL-c, high-density lipoprotein cholesterol; LDL-c, low-density lipoprotein cholesterol, SD, standard deviation

#### Prevalence of dyslipidaemia and other abnormal lipid levels

**Fig 1:**
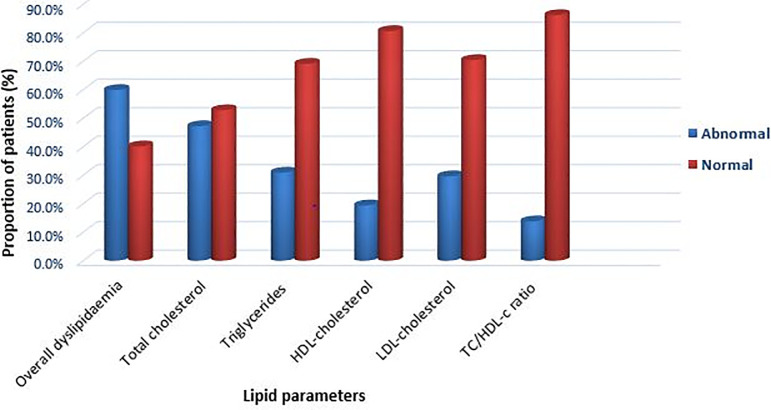
Prevalence of dyslipidaemia among HIV-infected patients receiving combination antiretroviral therapy.

Dyslipidaemia prevalence was 59.9% (95% CI 55.0–64.7%), and the main type of lipid abnormality was high TC (TC ≥ 200 mg/dl); which was found in 185 (47.3%) participants (Fig 1). High TG (TG ≥150 mg/dl) was found in 121 (30.9%) participants, low HDL-c (below 40 mg/dl) in 68 (19.4%), and high LDL-c (LDL-c ≥130 mg/dl) in 116 (29.6%) participants. Fifty-four participants (13.8%) had a high ratio of TC/HDL-c (TC/HDL-c ratio ≥ 5).

Table 3 shows the distribution of lipid abnormalities by types of ARV regimen. There were no significant differences in the prevalence of lipid abnormalities in patients on regimens that included TDF as compared with those using other NRTI–based regimens. The prevalence of lipid abnormalities was not different in the patients using NVP when compared with those using EFV–based regimen.

**Table 3:** Prevalence of lipid abnormalities among patients receiving ART by types of ART regimen.

ART regimens	TC ≥ 200 mg/dl	TG ≥ 150 mg/dl	HDL-c < 40 mg/dl	LDL-c ≥ 130 mg/dl	TC/HDL-c ratio ≥5
	n = 185 (%)	n = 121 (%)	n = 76 (%)	n = 116 (%)	n = 54 (%)
NRTI regimens					
TDF-based	81 (49.1)	49 (29.7)	30 (18.2)	53 (32.6)	24 (14.5)
AZT-based	76 (48.7)	50 (32.1)	32 (20.5)	45 (28.8)	21 (13.5)
D4T-based	28 (39.4)	22 (31.0)	14 (19.7)	18 (25.4)	9 (12.7)
***P*-value**	0.350	0.901	0.867	0.560	0.920
NNRTI regimens					
NVP-based	103 (47.9)	65 (30.2)	40 (18.6)	67 (31.2)	26 (12.1)
EFV-based	82 (48.3)	56 (31.6)	36 (20.3)	49 (27.7)	28 (15.8)
***P*-value**	0.755	0.764	0.666	0.453	0.287

ART, antiretroviral therapy; TC, total cholesterol; TG, triglyceride; HDL-c, high-density lipoprotein cholesterol; LDL-c, low-density lipoprotein cholesterol, NRTIs, nucleoside reverse transcriptase inhibitors; NNRTIs, nonnucleoside reverse transcriptase inhibitors; TDF, tenofovir; AZT, zidovudine; D4T, stavudine; EFV, efavirenz; NVP, nevirapine

#### Factors associated with abnormal lipid levels

Univariate and multivariable analysis were applied to assess factors associated with each lipid abnormality. All variables with univariate *P* values ≤ 0.25 were included in multivariable logistic regression analysis (ie, age, sex, residence, education, duration since HIV diagnosis, duration of ARV drug use, smoking, alcohol use, BMI and CD4 counts) (Table 4).

**Table 4:** Factors associated with abnormal lipid levels among HIV-infected patients receiving ART in North Shewa, Ethiopia.

Variables	TC ≥ 200 mg/dl	TG ≥ 150 mg/dl	HDL-c < 40 mg/dl	LDL-c ≥ 130 mg/dl
	Adjusted OR (95% CI)	Adjusted OR (95% CI)	Adjusted OR (95% CI)	Adjusted OR (95% CI)
Age (years)				
≤ 40	1	1	1	
> 40	**2.51 (1.64–3.84)**	**2.95 (1.85–4.71)**	**2.02 (1.17–3.50)**	**3.37 (2.08–5.47)**
*P*-value	**0.001**	**0.001**	**0.012**	**0.001**
Sex				
Male	NA	1.22 (0.75–1.98)	1.68 (0.97–2.94)	NA
Female	NA	1	1	NA
*P*-value		0.428	0.067	
Residence				
Rural	1	NA	1	1
Urban	1.48 (0.86–2.52)	NA	**2.61 (1.16–6.14)**	1.81 (0.95–3.44)
*P*-value	0.154		**0.021**	0.070
Education				
< High school	NA	1	NA	NA
≥ High school	NA	1.32 (0.81–2.16)	NA	NA
*P*-value		0.272		
Duration of infection (years)				
< 5	1	1	1	1
≥ 5	1.12 (0.56–2.24)	1.38 (0.61–3.18)	0.64 (0.18–2.25)	1.08 (0.45–2.57)
*P*-value	0.743	0.441	0.277	0.869
Duration on ART (years)				
< 5	1	1	1	1
≥ 5	1.54 (0.99–2.38)	**1.86 (1.13–3.07)**	**3.47 (1.75–6.80)**	**2.20 (1.30–3.71)**
*P*-value	0.050	**0.015**	**0.020**	**0.003**
Smoking				
No	1	1	1	1
Yes	1.83 (0.59–5.68)	2.85 (0.77–10.52)	**3.61 (1.06–12.34)**	1.81 (0.93–3.36)
*P*-value	0.292	0.117	**0.040**	0.383
Alcohol use				
No	1	1	NA	NA
Yes	0.51 (0.24–1.08)	0.89 (0.38–2.13)	NA	NA
*P*-value	0.078	0.808		
Body mass index (Kg/m^2^),				
≤ 25	1	1	1	1
> 25	**2.43 (1.19–4.97)**	**4.17 (2.01–8.67)**	2.04 (0.92–4.52)	**6.53 (3.05–13.98)**
*P*-value	**0.015**	**0.001**	0.078	**0.001**
CD4 count (cell/mm^3^)				
> 200	1	1	1	NA
≤ 200	**1.65 (1.05–2.58)**	0.81 (0.86–11.84)	0.58 (0.31–1.09)	NA
*P*-value	**0.031**	0.379	0.090	

TC, total cholesterol; TG, triglyceride; HDL-c, high-density lipoprotein cholesterol; LDL-c, low-density lipoprotein cholesterol; ART, antiretroviral therapy; NA, not available.

The multivariable logistic regression analysis revealed that older age (AOR = 2.51, 95% CI 1.64–3.84; *P* = 0.001), high BMI (AOR = 2.43, 95% CI 1.19–4.97; *P* = 0.015) and lower CD4 cell count (AOR = 1.65, 95% CI 1.05–2.58; *P* = 0.031) were significantly associated with higher TC. Older age (AOR = 2.95, 95% CI 1.85–4.71; *P* = 0.001), longer duration of ART use (AOR = 1.86, 95% CI: 1.13–3.07; *P* = 0.015) and high BMI (AOR = 4.17, 95% CI 2.01–8.67; *P* = 0.001) were associated with a high risk of raised TG. A lower HDL–c was independently associated with older age (AOR = 2.02, 95% CI 1.17–3.50; *P* = 0.012), living in an urban area (AOR = 2.61, 95% CI 1.16–6.14; *P* = 0.021), longer duration of ART use (AOR = 3.47, 95% CI 1.75–6.80; *P* = 0.020) and smoking (AOR = 3.61, 95% CI 1.06–12.34; *P* = 0.040). In addition, participants with older age (AOR = 3.37, 95% CI 2.08–5.47; *P* = 0.001), who had longer duration of ART (AOR = 2.20, 95% CI 1.30–3.71; *P* = 0.020), and high BMI (AOR = 6.53, 95% CI 3.05–13.98; *P* = 0.001) were more likely to have high LDL–c.

## Discussion

This study has demonstrated a high prevalence of dyslipidaemia (59.9%) in HIV-infected patients receiving ART at a hospital in Northeast Ethiopia. The most frequent form of dyslipidaemia was high TC (47.3%), followed by high TG (30.9%) and high LDL-c (29.6%). The overall prevalence of dyslipidaemia (59.9%) in this study was relatively lower than that reported in HIV-infected patients receiving first-line HAART in Southern Ethiopia (82.3%) [15]. It was also lower than those reported in HIV-infected patients receiving ART in the region, including 70.2% in rural Cameroon [16], 72.5% in Togo [17] and 77.5% in Dar es Salaam, Tanzania [18]; but higher than the 32.4% prevalence reported from Brazil [19]. The prevalence of high TC (47.3%) in our study was comparable with the prevalence rate reported among HIV patients on ART from Addis Ababa (42.1%) [20] and Southern Ethiopia (43.4%) [15], and from Tanzania (53.5%) [18]. However, it was higher than the prevalence reported from Southern Malawi (15.5%) [21] and rural Cameroon (29.8%) [16]. We found that the prevalence of high TG was 30.9%. This prevalence is lower than that reported from Addis Ababa [20] and Southern Ethiopia [15] among HIV patients who were on HAART (46.8 and 55.8%, respectively). This is also lower than the prevalence reported from rural Cameroon (51.8%) [16], but comparable to that of 28.7% reported from Malawi [21] and 29.6% from Tanzania [18]. The prevalence of raised LDL-c in our patients receiving ART (29.6%) was comparable to those reported from Southern Ethiopia (33.6%) [15] and Cameroon (33.3%) [16]. However, it was higher than the prevalence rate reported from Addis Ababa, Ethiopia (23%) [20], and lower than that of 59.8% reported from Tanzanian [18]. The prevalence of low HDL-c in our study was 19.4%, and this is comparable with the prevalence reported from Malawi (15.9%) [21], Tanzania (16.5%) [18] and Cameroon (18.4%) [16]. It was; however, lower than the prevalence reported from Addis Ababa (50.8%) [20] and Southern Ethiopia (43.4%) [15].

Nearly 14% of our participants on ART had a high TC/HDL-C ratio. Previous studies have reported prevalence of high TC/HDL-C ratio ranging from 16.7% to 45.1% in patients on standard combination first line ART [15, 16]. This finding has important implication as elevated TC/ HDL-c ratio may increase the risk of CVD [21]. A high TG/HDL-C ratio present in 3.7% of the patients on ART at a rural and an urban HIV clinic in Zomba district, Malawi was associated with all-cause mortality [9].

We found that older age was significantly associated with poor lipid profiles (TC, TG, LDL-c and HDL-c), and this was consistent with findings of previous studies [15, 18, 19, 22, 23]. In this and the above related studies, a significantly higher TC, LDL-c and TG levels, but lower HDL-c level was observed among older patients living with HIV which may cumulatively contribute to higher rates of cardiovascular disease. Thus, the diagnosis and management of lipid abnormalities in HIV-infected patients on standard combination first line ART are increasingly important with aging of this population. Although earlier study from Malawian adults on ART showed that lipid abnormalities were not significantly different between rural and urban patients on similar ART regimens [21], we found that living in urban areas is associated with lower plasma HDL-c levels. The observed alterations in the blood levels of HDL-c might be as a consequence of a less physically active life style in urban areas than those in rural settings perhaps due to more access to modern transportation systems and sedentary behaviours in the urban setting [24, 25]. HDL-c is a lipoprotein of cardiovascular protection and, it can be suggested that low levels of HDL-c may be significant factor of cardiovascular risk in urban patients on ART.

In the current study, being on ART for five or more years was associated with higher levels of TG and LDL-c but with lower levels of HDL-c. This is probably related to a combination of the effects of an aging HIV-infected population coupled with improved health due to suppression of viral load and the effect of ARV drugs on lipid metabolism. This was comparable to previous findings, which showed that long term exposure to ART was associated with poor lipid profiles [16, 18, 19]. Atherogenic serum lipid changes were observed among HIV-infected patients early after the initiation of ART, which may worsen over time [7]. A meta-analysis of data examining the impact of ART on the lipid profiles of people living with HIV worldwide also demonstrated that the risk of dyslipidaemia only emerges in patients on ART for no less than one year, and increases thereafter with prolonged treatment [26]. Patients with advanced HIV disease, i.e. lower CD4 lymphocyte count were more likely to have higher TC levels in our study. This is consistent with the results of Ceccato et al. [19]; who found that among patients with dyslipidaemia, most individuals had lower CD4 counts (< 200 cells/mm^3^). A study from a South African population on ART also reported unfavorable lipid profile changes, with lower CD4 count being risk factor for elevated cholesterol [27]. This is; however; in contrast with the previous reports involving patients on ART where lower CD4 count was associated with better lipid profile [15, 16, 18].

The present study revealed that patients with high BMI (≥ 25 kg/m^2^) were significantly associated with poor lipid profiles (raised TC, TG and LDL-c). This is in line with the findings of the study in Southern Ethiopia [15] and Tanzania [18] in which high BMI was significantly associated with lipid profile alterations in patients on ART. A study by Muya and Kamuhabwa [23], in Tanzania also reported a significant association between BMI of ≥ 25 kg/m^2^ with raised levels of TC and LDL-c. Changes in body fat distribution expected to occur with continued exposure to ART could offer a potential explanation for these findings. This study also found an association between smoking and decreased levels of HDL-c. Though the proportion of smokers in our sample population was very small and made up exclusively of men, the association is in agreement with previous reports, which indicated that ART-exposed smokers had poorer lipid profiles compared with non-smokers [16, 23, 26]. Given the effects of smoking on lipid levels and it’s contribute to the increased risk of cardiovascular disease endpoints among patients living with HIV, smoking cessation efforts should be made a priority in HIV care.

Our study found no significant differences in the prevalence of lipid abnormalities in patients on regimens that included TDF as compared with those using other NRTI–based regimens, which was similar to the findings in Southern Ethiopia [15], rural Cameroon [16], and Tanzania [18]. However, the Brazilian study found higher prevalence rate of dyslipidaemia among patients using NRTI-based regimens, especially when the regimen included stavudine (D4T) [19]. The non-TDF NRTIs regimens have been shown to increase TC in the South African population on first-line ART [27]. The literatures also presented data suggesting that treatment with NRTIs-based ART (stavudine, didanosine, zidovudine or lamivudine) has been frequently associated with lipid alterations, particularly lipoatrophy and hypertriglyceridemia [3, 5]. Similarly, no difference was observed in prevalence of lipid abnormalities between patients receiving NVP and those receiving EFV. The findings of this study are in line with other cross-sectional studies [15, 16, 18]. In contrast, treatment with efavirenz-based regimens were associated with a significantly higher risk of higher TC and TG levels than were nevirapine-containing NNRTI regimens [3, 28]. Therefore, further studies are required to identify the possible association between specific antiretroviral drugs and the development of lipid abnormalities in HIV infected patients.

### Limitations of the study

Our present study is limited by its small sample size, and lack of ART-naïve or HIV negative controls. Its cross-sectional nature also made it impossible to assume any causality. Well-controlled cohort studies would be appropriate to evaluate lipid profile alterations in patients using combination first-line ART regimen and their potential impact on cardiovascular health of people living with HIV in our settings.

## Conclusions

In conclusion, our study findings indicate a high prevalence of dyslipidaemia among HIV-infected patients receiving first-line ART. This study also identified some modifiable risk factors associated with abnormal lipid levels in the study population. There is a need for monitoring of lipid levels in patients with HIV on long term first-line ART with a special attention to be focused on older age, urban residence, longer duration of ART use, high BMI and smokers.

## Supporting information

S1 File Questionnaire for a study on prevalence and associated factors of impaired renal function and albuminuria among adult patients admitted to a hospital in Northeast Ethiopia, 2020. (DOCX)

## Abbreviations

AOR: Adjusted odds ratio
ART: antiretroviral therapy
BMI: body mass index
CI: Confidence interval
HDL-c: high-density lipoprotein cholesterol
LDL-c: low-density lipoprotein cholesterol
MMH: Mehal Meda Hospital
TC: total cholesterol
TG: triglyceride

## References

[1] Otieno G, Whiteside YO, Achia T, Kwaro D, Zielinski-Gutierrez E, Ojoo S, et al. Decreased HIV-associated mortality rates during scale-up of antiretroviral therapy, 2011–2016: a population-based cohort study. AIDS. 2019;33(15):2423–30. 10.1097/QAD.0000000000002374 31764107

[2] Troll J. Approach to dyslipidemia, lipodystrophy, and cardiovascular risk in patients with HIV infection. Curr Atheroscler Rep. 2011;13:51–6. 10.1007/s11883-010-0152-1 21181310 PMC3018260

[3] Calza L, Colangeli V, Manfredi R, Bon I, Re MC, Viale P. Clinical management of dyslipidaemia associated with combination antiretroviral therapy in HIV-infected patients. J Antimicrob Chemother. 2016;71:1451–65. 10.1093/jac/dkv494 26846208

[4] Estrada V, Portilla J. Dyslipidemia Related to Antiretroviral Therapy. AIDS Rev. 2011;13(1):49–56. 21412389

[5] Souza SJ, Luzia LA, Santos SS, Rondó PHC. Lipid profile of HIV-infected patients in relation to antiretroviral therapy: a review. Rev Assoc Med Bras. 2013;9(2):186–98. 10.1016/j.ramb.2012.11.003 23582562

[6] da Cunha J, Maselli LMF, Stern ACB, Spada C, Bydlowski SP. Impact of antiretroviral therapy on lipid metabolism of human immunodeficiency virus-infected patients: Old and new drugs. World J Virol. 2015;4(2):56–77. 10.5501/wjv.v4.i2.56 25964872 PMC4419122

[7] Riddler S, Li X, Chu H, Kingsley L, Dobs A, Evans R, et al. Longitudinal changes in serum lipids among HIV-infected men on highly active antiretroviral therapy. HIV Med. 2007;8:280–7. 10.1111/j.1468-1293.2007.00470.x 17561873

[8] Grover SA, Coupal L, Gilmore N, Mukherjee J. Impact of Dyslipidemia Associated With Highly Active Antiretroviral Therapy (HAART) on Cardiovascular Risk and Life Expectancy. Am J Cardiol. 2005;95:586–91. 10.1016/j.amjcard.2004.11.004 15721096

[9] Amberbir A, Banda V, Singano V, Matengeni A, Pfaff C, Ismail Z, et al. Effect of cardio-metabolic risk factors on all-cause mortality among HIV patients on antiretroviral therapy in Malawi: A prospective cohort study. PLoS ONE. 2019;14(1):e0210629. 10.1371/journal.pone.0210629 30653539 PMC6336397

[10] Richter A, Pladevall M, Manjunath R, Lafata J, Xi H, Simpkins J, et al. Patient characteristics and costs associated with dyslipidaemia and related conditions in HIV-infected patients: a retrospective cohort study. HIV Med. 2005;6:79–90. 10.1111/j.1468-1293.2005.00269.x 15807713

[11] Noubiap JJ, Bigna JJ, Nansseu JR, Nyaga UF, Balti EV, Echouffo-Tcheugui JB, et al. Prevalence of dyslipidaemia among adults in Africa: a systematic review and meta-analysis. Lancet Glob Health. 2018;6:e998–1007. 10.1016/S2214-109X(18)30275-4 30103999

[12] Dimala CA, Blencowe H, Choukem SP. The association between antiretroviral therapy and selected cardiovascular disease risk factors in sub-Saharan Africa: A systematic review and meta-analysis. PLoS ONE. 2018;13(7):e0201404. 10.1371/journal.pone.0201404 30059546 PMC6066235

[13] Friedewald WT, Levy RI, Fredrickson DS. Estimation of the concentration of low-density lipoprotein cholesterol in plasma, without use of the preparative ultracentrifuge. Clin Chem. 1972;18:499–502. 4337382

[14] Executive summary of the third report of the national cholesterol education program (NCEP) expert panel on detection, evaluation, and treatment of high blood cholesterol in adults (adult treatment panel III). JAMA 2001, 285(19):2486–97. 10.1001/jama.285.19.2486 11368702

[15] Tadewos A, Addis Z, Ambachew H, Banerjee S. Prevalence of dyslipidemia among HIV-infected patients using first-line highly active antiretroviral therapy in Southern Ethiopia: a cross-sectional comparative group study. AIDS Res Ther. 2012;9:31. 10.1186/1742-6405-9-31 23095661 PMC3499183

[16] Bekolo CE, Nguena MB, Ewane L, Bekoule PS, Kollo B. The lipid profile of HIV-infected patients receiving antiretroviral therapy in a rural Cameroonian population. BMC Public Health. 2014;14:236. 10.1186/1471-2458-14-236 24606888 PMC3973972

[17] Moukaila AR, Bawe LD, Mossi EK, Patassi AA, Tsevi YM, Nemi KD, et al. Dyslipidemia in People Living with HIV on Anti-Retroviral Treatment: Case of the Ambulatory Treatment Center (ATC) of the Sylvanus Olympio University Hospital of Lome. Open J Intern Med. 9:141–57.

[18] Ombeni W, Kamuhabwa AR. Lipid Profile in HIV-Infected Patients Using First-Line Antiretroviral Drugs. JIAPAC. 2016;15(2):164–71. 10.1177/2325957415614642 26514630

[19] Ceccato M, Bonolo P, Souza Neto A, Araújo F, Freitas M. Antiretroviral therapy-associated dyslipidemia in patients from a reference center in Brazil. Braz J Med Biol Res. 2011;44(11):1177–83. 10.1590/s0100-879x2011007500129 22052375

[20] Abebe M, Kinde S, Belay G, Gebreegziabxier A, Challa F, Gebeyehu T, et al. Antiretroviral treatment associated hyperglycemia and dyslipidemia among HIV infected patients at Burayu Health Center, Addis Ababa, Ethiopia: a cross-sectional comparative study. BMC Res Notes. 2014;7:380. 10.1186/1756-0500-7-380 24950924 PMC4077831

[21] Amberbir A, Singano V, Matengeni A, Ismail Z, Kawalazira G, Chan AK, et al. Dyslipidemia among rural and urban HIV patients in south-east Malawi. PLoS ONE. 2018;13(5):e0197728. 10.1371/journal.pone.0197728 29782548 PMC5962094

[22] Samuel M, Jose S, Winston A, Nelson M, Johnson M, Chadwick D, et al. The effects of age on associations between markers of HIV progression and markers of metabolic function including albumin, haemoglobin and lipid concentrations. HIV Med. 2014;15:311–6. 10.1111/hiv.12103 24245861 PMC4265250

[23] Muya E, Kamuhabwa A. Comparative Assessment of the Magnitude of Hyperlipidemia in HIV-Infected Patients Receiving Lopinavir/r- and Atazanavir/r-Based Antiretroviral Drugs. JIAPAC. 2019;18:1–10. 10.1177/2325958219841908 30995874 PMC6748546

[24] Romancini JLH, Guariglia D NN Jr, Herold P, Pimentel GG de A, Pupulin ÁRT. Levels of physical activity and metabolic alterations in people living with HIV /AIDS. Rev Bras Med Esporte. 2012;18(6):356–60.

[25] Dang AK, Nguyen LH, Nguyen AQ, Tran BX, Tran TT, Latkin CA, et al. Physical activity among HIV-positive patients receiving antiretroviral therapy in Hanoi and Nam Dinh, Vietnam: a crosssectional study. BMJ Open. 2018;8:e020688. 10.1136/bmjopen-2017-020688 29748343 PMC5950700

[26] Nduka C, Sarki A, Uthman O, Stranges S. Impact of antiretroviral therapy on serum lipoprotein levels and dyslipidemias: A systematic review and meta-analysis. Int J Cardiol. 2015;199:307–18. 10.1016/j.ijcard.2015.07.052 26241636

[27] Jamieson L, Evans D, Brennan A, Moyo F, Spencer D, Mahomed K, et al. Changes in elevated cholesterol in the era of tenofovir in South Africa: risk factors, clinical management and outcomes. HIV Med. 2017;18:595–603. 10.1111/hiv.12495 28332270

[28] Fontas E, van Leth F, Sabin C, Friis-Møller N, Rickenbach M, A d’Arminio Monforte, et al. Lipid Profiles in HIV-Infected Patients Receiving Combination Antiretroviral Therapy: Are Different Antiretroviral Drugs Associated with Different Lipid Profiles? JID. 2004;189:1056–74. 10.1086/381783 14999610

