# Supplementary material for: Prevalence of dyslipidaemia among HIV-infected patients receiving combination antiretroviral therapy in North Shewa, Ethiopia
Source: PLoS One. 2021 Apr 27;16(4):e0250328. doi: 10.1371/journal.pone.0250328 (PMC8078799; doi:10.1371/journal.pone.0250328)
Supplement: S1 File — (DOCX) [file pone.0250328.s001.docx]

**Questionnaire for a study on prevalence and associated factors of impaired renal function and albuminuria among adult patients admitted to a hospital in Northeast Ethiopia, 2020.**

| Identification No.__________________  PART 1- Questions Related to Socio-Demographic Characteristics | | | | | |
| --- | --- | --- | --- | --- | --- |
| \| Q. \|  \|  \| \| --- \| --- \| --- \| | QUESTION | | Check the Appropriate Option or Enter Numbers | | |
| 100 | What is the sex of the respondent? | | 1. Male  2. Female | | |
| 101 | What is the age of the respondent? | | Age in years (18-99) ________ (yy.m)  (write “99+”, if >99 years) | | |
| 102 | Residence of the respondent | | 1. Urban  2. Rural | | |
| 104 | \| Marital Status of the Respondent \| \| --- \| | | \| 1. Monogamy  2. Polygamy  3. Single \| 4. Widowed  5. Divorced \| \| --- \| --- \| | | |
| 105 | \| Ethnicity of the Respondent \| \| --- \| | | \| 1. Oromo  2. Gurage  3. Amhara \| 4. Tigre  5. Other(specify)_______ \| \| --- \| --- \| | | |
| 106 | What is the educational status of the respondent? | | \| 1. Illiterate  2. Grade1-8 \| 3. Grade9-12  4. Tertiary \| \| --- \| --- \| | | |
| 107 | Have you ever smoke cigarette in the past 12 month? | | 1.Yes | | 2. No |
| 108 | Alcohol intake (with frequency per week) | | 1. No  2. Once weekly | | 3. Twice weekly  4. Three or more |
| PART 2: Clinical and Laboratory Measurements | | | | | |
| 201 | Duration since HIV diagnosis | ______________ months | | | |
| 202 | Duration on ART | ______________ months | | | |
| 203 | Type of ART regimen | 1. TDF-3TC-NVP  2. TDF-3TC-EFV  3. AZT-3TC-NVP  4. AZT-3TC-EFV | | 5. D4T-3TC-NVP  6. D4T-3TC-EFV | |
| 204 | Weight | _____________kg (kilogram) | | | |
| 205 | Height | ____________cm (cent meter) | | | |
| 206 | CD4 count | _____________ cells/mm^3^ | | | |
| 207 | Serum lipid Levels | TC ___________mg/dl  TG ___________mg/dl  HDL-C ___________mg/dl | | | |
